# Complex restitution behavior and reentry in a cardiac tissue model for neonatal mice

**DOI:** 10.14814/phy2.13449

**Published:** 2017-10-09

**Authors:** Andreas Mayer, Philip Bittihn, Stefan Luther

**Affiliations:** ^1^ Max Planck Institute for Dynamics and Self‐Organization Göttingen Germany; ^2^ German Center for Cardiovascular Research (DZHK) Partner Site Göttingen Göttingen Germany; ^3^ Institute for Nonlinear Dynamics Georg‐August‐Universität Göttingen Göttingen Germany; ^4^ Institute of Pharmacology and Toxicology University Medical Center Göttingen Germany; ^5^ Department of Physics and Department of Bioengineering Northeastern University Boston MA USA; ^6^ Present address: Laboratoire de Physique Théorique CNRS Université Pierre et Marie Curie and École Normale Supérieure Paris France; ^7^ Present address: BioCircuits Institute University of California San Diego, La Jolla California

**Keywords:** Alternans, cardiac tissue, mathematical modeling, neonatal mice, reentry, restitution

## Abstract

Spatiotemporal dynamics in cardiac tissue emerging from the coupling of individual cardiomyocytes underlie the heart's normal rhythm as well as undesired and possibly life‐threatening arrhythmias. While single cells and their transmembrane currents have been studied extensively, systematically investigating spatiotemporal dynamics is complicated by the nontrivial relationship between single‐cell and emergent tissue properties. Mathematical models have been employed to bridge this gap and contribute to a deepened understanding of the onset, development, and termination of arrhythmias. However, no such tissue‐level model currently exists for neonatal mice. Here, we build on a recent single‐cell model of neonatal mouse cardiomyocytes by Wang and Sobie (*Am. J. Physiol. Heart Circ. Physiol*. 294:H2565) to predict properties that are commonly used to gauge arrhythmogenicity of cardiac substrates. We modify the model to yield well‐defined behavior for common experimental protocols and construct a spatially extended version to study emergent tissue dynamics. We find a complex action potential duration (APD) restitution behavior characterized by a nonmonotonic dependence on pacing frequency. Electrotonic coupling in tissue leads not only to changes in action potential morphology but can also induce spatially concordant and discordant alternans not observed in the single‐cell model. In two‐dimensional tissue, our results show that the model supports stable functional reentry, whose frequency is in good agreement with that observed in adult mice. Our results can be used to further constrain and validate the mathematical model of neonatal mouse cardiomyocytes with future experiments.

## Introduction

In the last two decades, an everincreasing number of detailed biophysical models of the ionic currents in ventricular myocytes underlying action potential (AP) generation have been developed (Fink et al. 2011; Winslow et al. 2011). Species‐specific models have been published for a number of species including guinea pig (Luo and Rudy 1991, 1994), rat (Pandit et al. 2001), mouse (Bondarenko et al. 2004; Wang and Sobie 2008; Koivumäki et al. 2009; Li et al. 2010), and human (ten Tusscher et al. 2004; Bueno‐Orovio et al. 2008) as it was found to be necessary to account for species‐specific differences in current contributions to the AP. More recently, important developmental changes in cardiac electrophysiology of mouse myocytes have been identified (Nuss and Marban 1994; Wang et al. 1996; Wetzel and Klitzner 1996; Wang and Duff 1997; Sabir et al. 2008; Wang and Sobie 2008; Korhonen et al. 2009; Kawamura et al. 2010). Neonatal ventricular myocytes of mice differ from their adult counterparts in a number of properties, including smaller size, greater currents through the Na^+^/Ca^2+^ pump reduced outward K^+^ currents, and reduced role of the SR to calcium release during a systole. The differences in currents lead to a longer, less spike‐like AP of neonatal cardiomyocytes compared to those of adult mice.

While single‐cell models have undoubtedly been a great success, there remains a gap between the dynamics in a single cell and tissue‐level dynamics in a real heart. Models of cardiac tissue can be built on top of existing single‐cell models by coupling many such cells with electrotonic currents dependent on the differences in membrane potential along the tissue (Clayton et al. 2011). The coupling of cells in tissue can qualitatively change the dynamic behavior of the cells due to electrotonic currents in a number of different ways (see References [Bueno‐Orovio et al. 2008; Clayton et al. 2011] and references therein for examples of the following): First, the AP shape can be altered. Most notably the action potential amplitude decreases in many tissue models in comparison to the corresponding single‐cell model. Furthermore, prolongations of the action potential duration (APD) have been reported in the literature for some models. Second, the restitution properties, that is, the rate adaptation of dynamical properties such as the APD or ionic concentrations, may change. Third, the existence or onset of alternans, which is a beat‐to‐beat alternation in AP dynamics, can be different in tissue compared to a single cell.

Tissue models furthermore allow the study of emergent properties and dynamics (Clayton et al. 2011). The propagation of an AP is characterized by the conduction velocity (CV) of the tissue, and is dependent on both the diffusivity and the steepness of the AP upstroke. In tissue, alternans can be spatially concordant or discordant (De Diego et al. 2008), that is, the whole tissue alternating in phase or different regions alternating out‐of‐phase, respectively. Cardiac reentry, which occurs when a propagating pulse reexcites the heart after the refractory period has ended, is a mechanism producing cardiac arrhythmias. Spiral waves of electrical activity are hypothesized to explain cardiac reentry (Davidenko et al. 1992) and can only be directly studied in tissue models (Fenton and Karma 1998). The richness in dynamical behavior, not all of which can be seen in single cells, makes the study of tissue models essential for a detailed understanding of abnormal heart rhythms. The development of tissue models from single‐cell models, although, has not always yielded sensible results. The difference between the electrotonic currents in tissue and the stimulus current used in single‐cell models may cause unphysiological change in transmembrane currents (Cherry and Fenton 2007; Clayton et al. 2011). A careful analysis of the dependence of the AP and its underlying currents on the kind of stimulation is thus necessary for the validation of tissue models.

**Figure 1:**
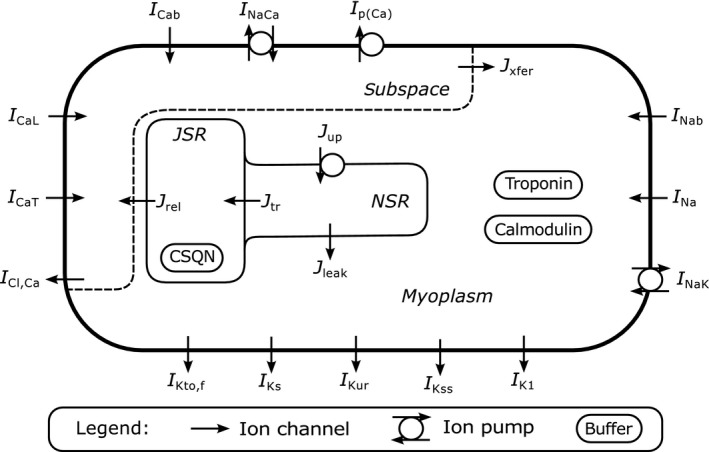
Sketch of the components of the Wang–Sobie model. The model distinguishes 14 sarcolemmal currents and describes intracellular calcium cycling between different model compartments (myoplasm, subspace, junctional sarcoplasmic reticulum [JSR], network sarcoplasmic reticulum [NSR]). The calcium concentrations are buffered by troponin, calmodulin, and calsequestrin [CSQN].

Despite the use of neonatal mice as an experimental model system, to our knowledge, no computational studies of cardiac tissue of developing mice have been reported in the literature. The current work thus presents a model of neonatal mouse ventricular tissue and characterizes its dynamical properties. Our model for the ventricular tissue of neonatal mice builds on a single‐cell model published recently (Wang and Sobie 2008). The model was the first to incorporate data on developmental changes in mice and was built as a modification of an earlier model of adult mouse cardiomyocytes (Bondarenko et al. 2004). It was fit to match data from day 1 neonatal cells (especially (Wang and Duff 1997; Wang et al. 1996)) and includes a wide range of ionic channels, pumps, and exchangers as well as detailed intracellular calcium cycling (Fig. 1). A computational model of neonatal mouse ventricular tissue will be able to make predictions for the dynamics that occur in the actual mouse heart, as well as in artificial multicellular substrates (Ralphe and de Lange 2013), for example, monolayers of cultured neonatal mouse myocytes. Cell cultures with different artificially imposed geometries are important experimental systems for research on cardiac electrophysiology and have been used for neonatal cardiomyocytes from a number of species including rat (De Diego et al. 2008; Majumder et al. 2016) and mouse (Thomas et al. 2000; Richter 2011). Another reason why mice are important model animals is the possibility of using genetically modified mice to study the effects of, for example, ion channel mutations (Thomas et al. 2003). Therefore, a tissue‐level model of neonatal mouse ventricular tissue might enable predicting the influence of genetic modifications on the spatiotemporal dynamics in tissue or corresponding culture systems.

## Methods

### Model development

To enable the model cardiomyocytes to reach stable limit cycle oscillations and maintain homeostasis during prolonged periods of external stimulation, we introduced two modifications to the original single‐cell model. First, the stimulus current was attributed to the influx of potassium ions. Second, the calcium‐activated chloride current *I*_Cl,Ca_ was removed as it prevented homeostasis through a net outward flux of ions at every AP. Both changes are discussed in detail in Section III A. The description of the other channels and of the intracellular calcium handling was left unchanged.

Using a continuum approximation of isotropic cardiac tissue, we described the coupling of cells by the monodomain equation of cardiac tissue (Clayton et al. 2011).

(1) $$\partial_{t}V\left(t,\vec{x}\right)=D\nabla^{2}V\left(t,\vec{x}\right)-I_{ion}\left(t,\vec{x}\right)$$

where *V* (*t,* $\overset{¯}{x}$) is the membrane potential at time *t* and position $\overset{¯}{x}$, *D* is a diffusivity describing the electrical conductivity of the tissue, and *I*_*ion*_(*t,* $\overset{¯}{x}$) is the sum of all sarcolemmal currents, which are calculated from the single‐cell model. Note that we express all ionic currents as current per membrane capacitance in units of pA/pF. As potassium is by far the most abundant ion species inside cardiomyocytes, we assumed that the electrotonic current is carried by potassium ions. This assumption has also been made in other models of cardiac tissue (ten Tusscher et al. 2004; Bueno‐Orovio et al. 2008). The diffusivity *D* was set to 1 cm^2^/s to reproduce the CVs, which were observed in strands of cultured neonatal mouse cardiomyocytes (Thomas et al. 2000). The diffusivity can also be estimated from the surface‐to‐volume ratio *S*_*V*_, the specific membrane capacitance *C*_*m*_, and the resistivity *ρ* of a cardiomyocyte as (Bueno‐Orovio et al. 2008) *D *=* *1/(*S*_*V*_ *ρC*_*m*_). A lumped resistivity taking into account both cytoplasmic and junctional effects of *ρ *= 203 Ω cm has been reported for cultured neonatal mouse cardiomyocytes (Thomas et al. 2003). Inserting this resistivity and the specific capacitance and cell geometry assumed in the single‐cell model into this equation yields a diffusivity (*D *=* *1.01 cm^2^/s) in good agreement with the value chosen above. Note that the choice of diffusivity does not affect the type of behavior observable in tissue, but only acts to rescale the spatial length scale.

The complete set of equations, parameters and initial conditions defining the model can be found in Appendix S1.

### Numerical methods

Sequential splitting was used to decompose the time stepping into integrating the single‐cell dynamics and solving the spatial diffusive coupling (Csomós et al. 2005; Press et al. 2007). For solving the diffusive coupling of cells in tissue, the spatial domain was discretized uniformly with a grid spacing *h *=* *0.015 cm. On this grid, the diffusion was calculated using a forward time‐entered space finite difference method with a standard Laplace kernel (Press et al. 2007). In all simulations, no‐flux boundary conditions were used, that is, the normal derivative of the membrane potential at the boundaries was assumed to be zero. The system of ordinary differential equations describing a single cell was time stepped with the adaptive Runge–Kutta (Bondarenko et al. 2004; Bueno‐Orovio et al. 2008) method from the GNU Scientific Library (Galassi et al. 2002). The adaptive time stepping used relative and absolute error tolerances of 10^−6^ and 10^−16^, respectively, with a maximal step‐size of 0.01 ms. The correctness of the implementation was checked by comparing the time course of all dynamical variables during a simulated action potentials with a reference implementation by (Wang and Sobie 2008). To assess the influence of the discretization, propagation of a traveling pulse was analyzed with doubled spatial resolution and quadrupled temporal resolution. The conduction velocity differed by <5%, which is commonly considered acceptable convergence (Clayton et al. 2011).

### Simulation protocols and analysis methods

To induce cardiac excitation in our simulations, we use a stimulus current *I*_stim_ = *−*80 pA/pF for a duration of 0.5 ms. The strength of the stimulus current corresponds to approximately two times the stimulation threshold of a quiescent cell. To simulate the adaptation of the cells to different pacing frequencies, we applied a long series of stimuli at fixed time intervals, the basic cycle length (BCL). As a measure of the length of an AP, we calculated APDs as the time interval between crossings of a threshold voltage of *−*70 mV. This definition of APD corresponds to the APD at about 90% repolarization for 1 Hz pacing.

We simulated the dynamics of the tissue model in an one‐dimensional cable of 5 cm length and in a two‐dimensional sheet of tissue 5 *×* 5 cm in size. In the cable, propagating action potentials were started by applying the same stimulus current as in single‐cell simulations to the first 0.1 cm of the cable. To study reentry dynamics in the two‐dimensional sheet of tissue, all dynamical variables were initially set to a value obtained upon steady‐state pacing in a single cell except for the inhibitory gating variables (*h* and *j*) of the Na^+^‐channel, which were set to zero in the upper half of the simulation domain. The single‐cell stimulus current was then applied to the right half of the domain to excite activation. This method initiates spiral waves encircling a functional obstacle near the center of the simulated area.

The periodicity of the initiated spiral wave was studied by determining the time intervals between subsequent crossings of the threshold voltage *−*70 mV for each cell. The time intervals were calculated for all computational cells and then plotted as a histogram. To assess spiral wave meander, the movement spiral wave core was analyzed. The position of the spiral wave tip was defined by the intersection of the *V *= *−*50 mV isopotential line and the *∂*_t_ *V *= 0 contour line (Fenton and Karma 1998). The partial derivative was approximated by finite differences with ∆*t *=* *0.5 ms.

## Results

### Effects of long‐term pacing

Long‐term pacing at a fixed frequency is a part of many experimental protocols and is commonly used to determine steady‐state initial conditions in computational models (Bondarenko et al. 2004; Wang and Sobie 2008). Models which explicitly track the changing intracellular ionic concentrations as does the Wang–Sobie model, possess a great amount of memory, that is, their dynamical behavior is dependent on the series of previous excitations. Due to the effects of such memory on dynamical behavior, a proper choice of initial conditions is particularly important for these models to ensure that the model output does not depend on some unphysiological initial condition.

**Figure 2:**
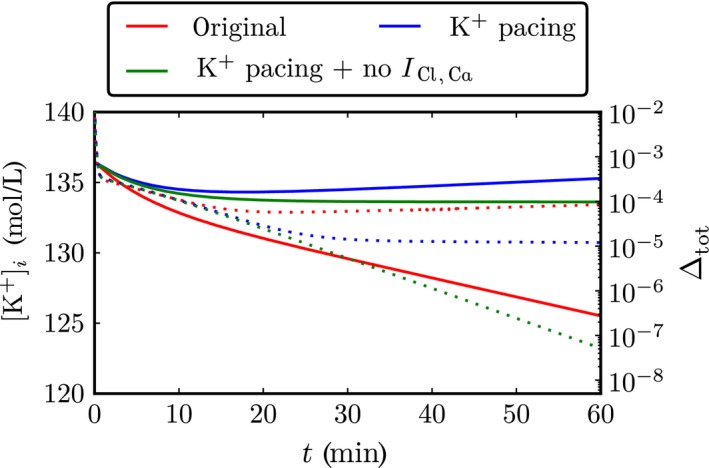
Effects of long‐term pacing: The original model drift upon pacing at 1 Hz is eliminated by conservative stimulation and removal of *I*_Cl,Ca_. The time course of the intracellular potassium concentration (solid lines) and of an aggregate measure of drift ∆_tot_ (dotted lines) is shown.

For the Wang–Sobie model, Wang and Sobie (2008) have given initial conditions, which are described as steady‐state values for a pacing frequency of 0.5 Hz. In trying to obtain steady‐state values for other frequencies, we discovered, however, that the dynamical variables drift during pacing at a fixed BCL and no steady state can be reached (Fig. 2, solid red line). The overall change in intracellular ion concentrations due to this drift can be substantial, as demonstrated by the about 10 mm drift in intracellular potassium concentration during simulated 60 min of long‐term pacing at 1 Hz. While Figure 2 shows results for a pacing frequency of 1 Hz, we note that similar results were obtained at other pacing frequencies including the pacing frequency of 0.5 Hz used in the original study (Fig. S3). The drift is even more pronounced for faster pacing frequencies (Fig. S4).

We found two origins to this drift: first, a nonconservative pacing is used that only changes the membrane potential without being attributed to any current; second, an outward chloride current is included in the model without any balancing inward chloride ion currents. To eliminate the drift, we modified the single‐cell model by attributing the pacing to a potassium current (Hund et al. 2001) and by eliminating the outward chloride current. These modifications allow the model to reach a stable steady state upon prolonged pacing (Fig. 2, solid green line). The larger contribution to the drift comes from the nonconservative pacing (Fig. 2, solid blue line) but both modifications are necessary to eliminate drift entirely. The removal of the chloride current *I*_Cl,Ca_ resulted in a very small change in the AP (Fig. S1). Using these modifications, steady‐state conditions could be obtained for every BCL used in a simulation.

Judging convergence from a visual inspection of changes in ion concentrations over time is difficult as shown by the near leveling off of potassium concentration with conservative pacing but without removal of the chloride current (Fig. 2, solid blue line). Therefore, we propose a more powerful numerical measure for convergence. Assessing convergence is particularly complicated by the fact that we do not know the final steady‐state value a priori. To get around this problem, we can instead look at what amounts effectively to a first derivative: the difference in each dynamical variable *q*_*i*_(*t *+ *τ)* *− q*_*i*_(*t*) between two snapshots of the system at stroboscobic intervals *τ,* which are a multiple of the BCL with which the system is driven. As the time constants of convergence along different directions in state space might be considerably different ideally, we should look at convergence for the different state variables. To define a scalar measure ∆_tot_ of overall drift, we combine all the differences across the *N* state variables,

(2) $$\Delta_{tot}=\sqrt{\frac{1}{N}\sum_{i=1}^{N}{\left(\frac{q_{i}\left(t+\tau \right)-q_{i}\left(t\right)}{\overset{¯}{q_{i}}}\right)}^{2}}$$

where we have rescaled the dynamical variables by their mean values $\overset{¯}{q_{i}}$ such that they all fall within a common range. As we get closer to steady state, ∆_tot_ should approach zero exponentially as the largest time constant will dominate. Acceptable convergence can then be defined using some cut‐off on ∆_tot_. In practice, we used ∆_tot_ *< *10^−7^ to ensure the convergence to steady state in the simulations presented in the following. The usefulness of this measure is demonstrated by how clearly it distinguishes between the nonconverging model with conservative pacing only and the converging model with conservative pacing and without the chloride current (blue and green dotted line in Fig. 2).

### Action potentials in single cells and tissue

Due to the influence of electrotonic currents, the AP and its underlying currents are known to differ between a stimulated single cell and a cell in tissue. To investigate these changes, we compared the single‐cell AP to the AP in a 5 cm one‐dimensional cable which was stimulated at one end. In the cable, we analyzed the AP in a cell in the middle of the cable, far away from the boundary, to avoid distortions of the membrane potential, and subsequently, the dynamics of all local variables, by electrotonic loading effects (Cherry and Fenton 2011).

A one‐dimensional cable provides a computationally efficient model system for the propagation of APs. Propagation of APs in a cable is equivalent to the propagation of planar waves in isotropic three‐dimensional media, because no diffusion transversal to the direction of propagation takes place for a planar wave. The quiescent steady state of an unpaced cell was used as the initial condition for the single cell and the cells in the cable. The quiescent steady state is the same in tissue and in single cells, which helps to clearly delineate differences directly due to electrotonic currents. During prolonged stimulation, further differences between single cells and tissue will emerge as a consequence of the different steady‐state values of the dynamical variables arising from the difference between stimulus and electrotonic current. Secondary differences due to coupling in tissue will turn out to play an important role for the emergence of alternans below.

**Figure 3:**
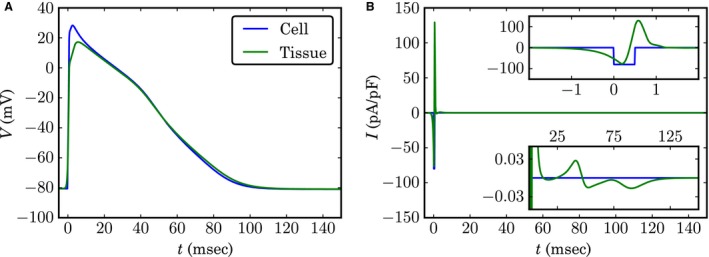
(A) Action potential (AP) in a single cell and in tissue. Time *t *= 0 ms was defined as the time point, when the upstroke surpasses *−*60 mV to align the two APs. (B) Time course of the stimulus current (single cell) and the electrotonic current (tissue). The upper inset shows a magnified view of the fast dynamics at the start of the action potential (AP), whereas the lower inset shows the small amplitude electrotonic currents during repolarization.

While the overall AP shape is similar in tissue and single cells, some differences were observed (Fig. 3A). The most visible difference is the reduction in APA from 109 mV to 98 mV (−10%). The maximal upstroke velocity $\frac{dV}{dt}{|}_{max}$ also decreases markedly from 213 mV/ms to 146 mV/ms (−32%). In contrast, the APD is only prolonged by 3% from 82  to 85 ms.

The changes in the AP can be understood by contrasting the stimulus current used in single‐cell simulations with the electrotonic current (Fig. 3B). The electrotonic current has a complex, biphasic time course that originates from the passage of the sharp action potential upstroke, while its contribution during comparatively slow repolarization is small. In contrast, the stimulus current is a simple step function to −80 pA/pF hold for 0.5 ms (see Section II C). The APA and $\frac{dV}{dt}{|}_{max}$ decrease, which can be explained as a consequence of the inversion of the electrotonic current from an inward to an outward current.

The alterations to the AP in tissue influence the dynamics of the voltage‐gated channels (Fig. S5). The most notable difference is *I*_*Na*_, which inactivates markedly slower. This increases the integrated flux through this channel by 30% from *−*0.064 pC/pF to *−*0.083 pC/pF, counteracting the effect of the increased outward electrotonic current.

### Restitution and alternans

As the spatiotemporal dynamics in tissue is known to be dependent on the BCL, we studied the effect of changing it in cells and in tissue.

We started by determining the APD restitution curve of the single‐cell model, as no description of restitution properties of the Wang–Sobie model can be found in the literature. Steep restitution curves have been linked to the genesis of alternans (Nolasco and Dahlen 1968; Cherry and Fenton 2004).

Due to memory effects, the APD is not only dependent on the preceding DI, but also on the pacing history. For determining restitution curves, a long series of pulses was thus first applied at a BCL S1 until convergence to a steady state was reached. The cell, which now had a well‐defined pacing history, was then stimulated after a time interval S2 and the resulting APD was measured. Two different types of restitution curves, S1–S2 restitution and dynamic restitution, were determined. The S1–S2 restitution curves describe the change in APD resulting from varying S2 for a fixed S1, whereas the dynamic restitution curve describes the relationship between BCL and APD at steady‐state conditions.

**Figure 4:**
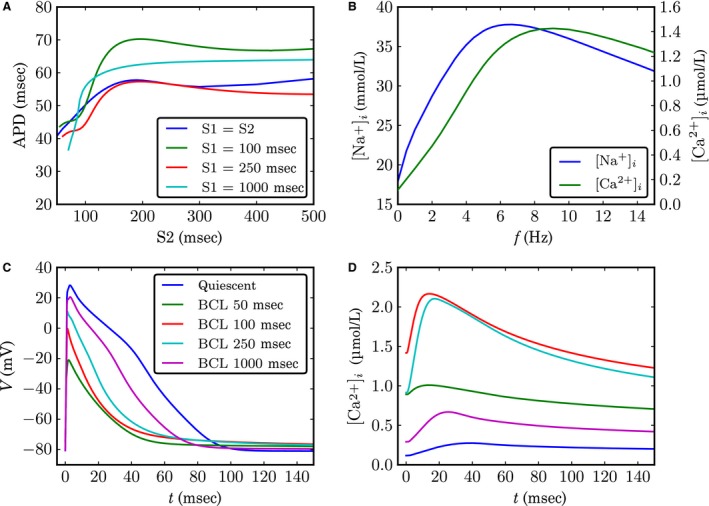
(A) action potential duration (APD) as a function of basic cycle length (BCL). Three examples of S1–S2 restitution curves (fixed S1, varying S2) and the dynamic restitution curve (S1 = S2) is shown. (B) Diastolic sodium and calcium concentration at steady state as a function of the pacing frequency. (C,D) Membrane potential and intracellular calcium concentration, respectively, during different steady‐state action potentials (APs).

The model displays a complex restitution behavior (Fig. 4A). The dynamic restitution curve shows a nonmonotonic dependence on BCL. It has a positive slope for short BCLs, which then changes into a small negative one from 180 ms up until a local minimum at 300 ms. The three examples of S1–S2 restitution curve are similar in that they all show APD shortening at small S2. The curve at a S1 interval of 1000 ms, however, differs in shape from the curves for S1 intervals of 100 ms and 250 ms in that it is monotonic with respect to S2. The biggest absolute differences in APDs between the S1–S2 curves are displayed at large BCLs. The AP at these BCLs is longer for S1 = 1000 ms compared to S1 = 250 ms. Surprisingly, this trend is reversed when increasing the pacing frequency further by going to S1 = 100 ms, where the APD even exceeds the APD for S1 = 1000 ms for most BCLs. Only the slope of the S1 = 1000 ms restitution curve did exceed 1, which is a commonly used predictor for alternans (Nolasco and Dahlen 1968). In line with the shallow dynamic restitution curve, no alternans was observed in single cells at steady state.

The pacing frequency also has a big influence on intracellular ion concentrations (Fig. 4B), the AP shapes (Fig. 4C), calcium transients (Fig. 4D) and the relative contribution of different transmembrane currents to the AP (Fig. S6). The diastolic intracellular calcium and sodium concentrations are higher during pacing than at rest, reaching a maximum at a pacing frequency of 6 Hz and 9 Hz, respectively. The APA decreases steadily with pacing frequency from 101 mV at 1 Hz to 77 mV at 10 Hz (−24%). Calcium transient amplitudes increase with pacing frequency up to S1 = 250 ms, but then decrease again for shorter S1 intervals.

To characterize the dynamics in the tissue model, we also conducted a restitution study in a cable. After initializing the cable with single‐cell steady‐state values for a pacing frequency of 0.5 Hz, it was continuously stimulated at one end at a fixed BCL. Subsequently, the properties of the 45th and 46th AP were analyzed. Steady state has not been reached by this time, and the restitution curve is thus not a dynamical restitution curve for tissue, but rather mimics some experimental restitution protocols without full adaptation to the S1 BCL.

**Figure 5:**
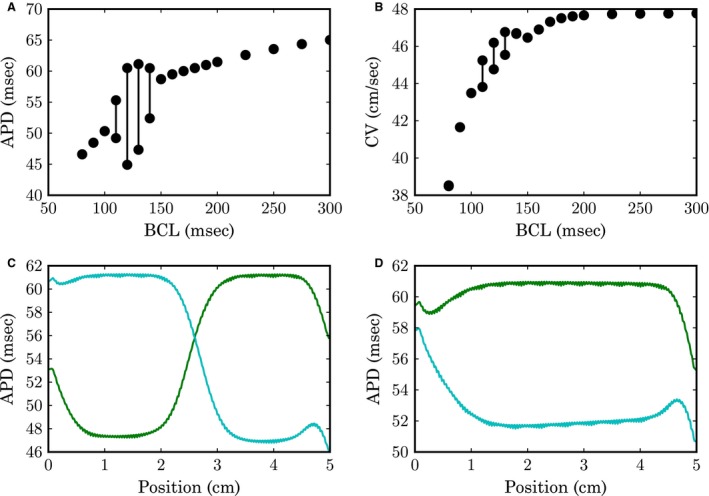
Restitution in a cable. Properties of action potential (AP) #45 and 46 after start of stimulation are shown for each basic cycle length (BCL). (A) Action potential duration (APD) measured at a cell 1 cm from the left of the cable. (B) CV measured between 1 and 2 cm from the left of the cable. (C) Spatial variations in APD at BCL = 130 ms. (D) Spatial variations in APD at BCL = 140 ms.

The APD reduces steadily for the BCLs which were studied (Fig. 5A), except for a region between a BCL of 110 ms and 140 ms, where alternans in APD were observed. The amplitude of the alternans was largest for a BCL of 120 ms, where the difference in APD amounted to 16 ms.

In tissue, the pacing frequency also has an influence on the CV (Fig. 4B). While the CV remained fairly constant up to a BCL of 200 ms, it then started to decline eventually reaching 38.5 cm/s at a BCL of 80 ms, which represents a decrease of −19% in comparison to the value at 300 ms.

The dynamics at two BCLs, at which alternans were observed, was analyzed more closely by calculating the APDs for every cell in the cable (Fig. 5C,D). At a BCL of 140 ms, all cells in tissue alternated in unison. After lowering the BCL by 10 ms, the alternans became spatially discordant, with two regions of the cable alternating out‐of‐phase.

### Spiral wave reentry

**Figure 6:**
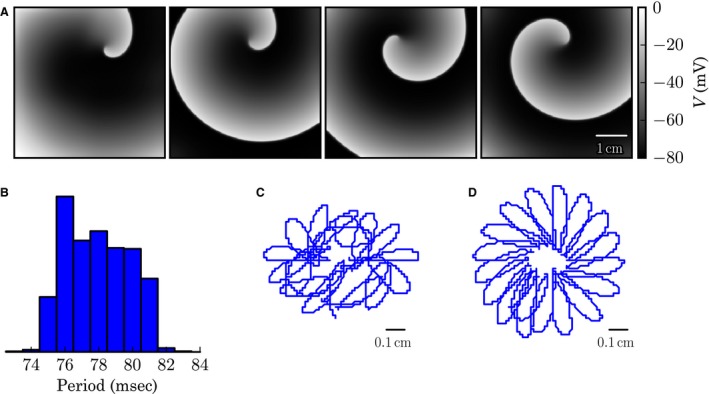
Spiral wave dynamics. (A) Snapshots of the membrane potential at time *t*/s *∈* {0.25*,* 1.0*,* 2.5*,* 5.0} (from left to right). (B) Histogram of the periods of the electrical activity during *t*/s *∈* [1.0*,* 2.0]. (bin size 1 ms). (C) Meandering of the spiral core for *t*/s *∈* [1.0*,* 2.5]. (D) Meandering of the spiral core for *t*/s *∈* [3.5*,* 5.0].

Stable spiral waves exist in our model of neonatal mouse cardiac tissue. Spiral waves could be initiated with a cross‐field method in a tissue, which was initialized with single‐cell steady‐state values obtained at 0.5 Hz pacing. During a simulation of 5 s length, the spiral wave rotated steadily without breaking up or visibly changing shape (Fig. 6A). To assess the properties of the spiral wave, its periodicity (Fig. 6B) and the movement of its tip were analyzed (Fig. 6C). The mean period of the membrane potential changes between second 1 and 2 after initiation of the spiral wave was 77.9 ms corresponding to a frequency of 12.8 Hz. There is a small dispersion in the periods on the order of a few milliseconds (Fig. 6B). The dispersion might partially be due to the meandering of the spiral core. The meander pattern is relatively irregular at the beginning of the simulation (Fig. 6C). After some time, however, the movement becomes increasingly regular (Fig. 6D). The meander pattern consists of outward petals arranged around a circular structure of about 0.15 cm in diameter.

## Discussion

### Model development

Models describing the discrete nature of the propagation of cardiac action potentials through gap junctions have been developed, but the usage of a continuum approximation of cardiac tissue remains widespread (Sneyd and Keener 2001; Clayton et al. 2011). A common continuum representation of cardiac tissue is the monodomain equation. The monodomain equation can be derived under the assumption of equal anisotropy of intra‐ and extracellular space from the bidomain equations, which describe tissue as being comprised of overlapping and continuous intra‐ and extracellular domains separated by the cell membrane.

We have developed a model of neonatal mouse ventricular tissue based on a single‐cell model and a description of electrotonic coupling of cells through the monodomain equation.

During the development of the tissue model, we noted deficiencies in the single‐cell model causing a drift of the model during repetitive stimulation. We have identified two reasons for this drift: Nonconservative stimulus currents and an inadequate description of transmembrane chloride currents. We note that generally in models which track intracellular ion concentrations homeostasis is only attainable if all changes to the membrane voltage are accounted for by fluxes of these ions. As the dynamical equations implement charge conservation, net voltage changes over a cycle through other currents can only be balanced by changes in the intracellular concentrations of the tracked ions leading inevitably to drift. Our study calls attention to this feature of such models, which seems to be underappreciated in the literature.

While nonconservative stimuli might, in fact, be a correct description in some experimental situations, they lead to highly undesirable consequences in computational models. As models with nonconservative stimulation cannot be driven to a steady state, setting initial conditions becomes problematic. Furthermore, no long‐term simulations can be performed at higher pacing rates, as the dynamical variables of the cell will quickly leave their physiological range under such conditions (Fig. S4). Following a proposal of Hund et al. (2001), we have therefore attributed the stimulus current to potassium fluxes, which is done in many of the more recent models of cardiac cells (Kneller et al. 2002; ten Tusscher et al. 2004; Bueno‐Orovio et al. 2008).

There is just one chloride current, the calcium‐activated chloride current *I*_Cl,Ca_, contained in the model. As it is net outward during a typical AP (Fig. S1), it breaches cellular homeostasis. Another modeling study tried to address a similar problem in a model of the canine atrial AP by incorporating an additional chloride background current and an empirically formulated Na^+^/Cl^*−*^ cotransporter (Kneller et al. 2002). We have opted against such an approach, as we felt there was not enough experimental data on murine neonatal chloride currents to parameterize such channels. Furthermore, if anionic fluxes are included in a model, the cells osmolarity may change during prolonged pacing, which is problematic in a model that does not account for volume changes (Fraser and Huang 2007). The elimination of *I*_Cl,Ca_ resulted in a small change in the AP, which is in agreement with experiments (Xu et al. 2002).

Both the authors of the Bondarenko model, which suffers from the same nonconvergence problems (Koivumäki et al. 2009), and of the Wang–Sobie model claimed that the initial condition represented a steady state during pacing. This highlights the need for a more careful and methodological assessment of convergence, as a properly defined steady state is required for the analysis of many dynamical phenomena such as alternans (Livshitz and Rudy 2007). We believe that introducing a scalar measure of drift, such as ours or similar ones reported in the literature (Livshitz and Rudy 2009), can be helpful in getting a quick overview about the convergence of high‐dimensional models.

The modeling of the electrotonic currents in tissue with the monodomain model is very widespread in the literature (Alonso et al. 2016), even though other models of tissue have been constructed. One limitation of the current study is that we have only considered isotropic and homogeneous tissue, whereas in the heart fibers introduce anisotropy, and fibroblasts and blood vessels introduce inhomogeneities (Clayton et al. 2011). These modeling assumptions might best be met by cultures of neonatal mouse myocytes, which are grown on unpatterned slides. Even these cell cultures, however, tend not to be perfectly homogeneous due to cell‐to‐cell variability and fibroblasts present in the culture and could be incorporated in the future in order to directly match such experiments, as was done in the case of monolayers of rat atrial cardiomyocytes (Majumder et al. 2016).

### Spatiotemporal dynamics in tissue

Using our newly constructed tissue model, we studied spatiotemporal dynamics in tissue and contrasted it to the single‐cell dynamics.

In our modeling process, we incorporated a single‐cell model as a source term for ionic currents into the monodomain equation. While this step is conceptually straightforward, unphysiological changes in the AP or in some transmembrane currents may result from switching from the single‐cell stimulus current to electrotonic currents in tissue as has been reported for some models (Cherry and Fenton 2007; Clayton et al. 2011). We have thus carefully analyzed differences between an AP in a single cell and in tissue. While there were differences in the AP shape, most notably a decreased APA and upstroke velocity in tissue compared to a single cell, the time course of most transmembrane currents changed little and the APD was essentially unchanged. The decreased upstroke velocity is at the lower end, but still in the range of cell‐to‐cell variability of upstroke velocities experimentally observed in day 1 neonatal ventricular mouse myocytes (Wang et al. 1996) and strands of cultured neonatal mice (Thomas et al. 2000).

The single‐cell model exhibits a complex restitution behavior with relatively shallow slopes of the restitution curves. The restitution curves obtained by the S1–S2 curve differed markedly demonstrating the effect of the memory. The diastolic intracellular calcium and sodium concentrations show a nonmonotonic dependence on pacing frequencies.

No alternans were observed in isolated cells at steady state. In cable simulations, alternans developed for some BCLs, and were either spatially concordant or discordant. To determine whether these alternans are indeed the result of cell‐to‐cell coupling, we performed control simulations in a single cell that replicate the pacing protocol in the cable as closely as possible. Starting at a converged 0.5 Hz steady state, we analyzed the APDs of all subsequent APs for BCLs that led to alternans in the cable (Fig. S2). While the APD decreased markedly with time as a result of rate adaptation, no alternating APDs were observed for any of the subsequent APs. While tissue‐level effects have previously been considered in other models as a factor that quantitatively changes alternans characteristics such as onset frequency and magnitude (Clayton et al. 2011), we conclude that, in this model, electrotonic coupling is what creates alternans in the first place (cf. Fig. 3B). Another finding of our numerical simulations is the existence of spiral waves that were stable during 5 sec of simulation. In contrast to observations of stationary reentry in the adult mouse heart (Vaidya et al. 1999), the spiral tip meandered on paths which became more regular with time. While the simulation domain was clearly larger than the ventricular surface area of a neonatal mouse heart, it should be possible to compare this prediction with monolayers of cultured neonatal mouse cardiomyocytes. Note that the boundary conditions might have an influence on the meander pattern of the spiral due to loading effects (Cherry and Fenton 2011), so it might be necessary to adapt the geometry to the specific experimental situation. Nevertheless, in our case, the dominant frequency of the spiral wave was 12.8 Hz which is in good agreement with the frequencies of around 13.6 Hz seen during ventricular tachycardia in adult mouse heart (Vaidya et al. 1999).

In summary, we have constructed and explored a mathematical model for ventricular tissue of neonatal mice. To do this, we modified an existing single‐cell model, such that it displays physiological behavior for typical pacing experiments used to characterize cardiac tissue, and added spatial interaction to simulate the propagation of action potentials through one‐dimensional cables and in tissue. Matching higher order properties such as APD and CV restitution curves and the dynamics of spiral wave reentry with experimental data is indispensable in order to correctly capture the emergence and stability of arrhythmias, which, together with determining their underlying dynamic and genetic causes, is one of the primary reasons for the study of cardiac electrophysiology. Our results yield a number of experimentally testable predictions and can thus be used to provide additional layer of validation that will lead to a refinement of the underlying single‐cell model of neonatal mouse cardiomyocytes. In addition, our results can serve as the base line for the incorporation of additional spatial structure introduced by tissue geometry, fibrotic tissue, and cell‐to‐cell variability.

## Conflict of Interest

None declared.

## Data Accessibility

## Supporting information




**Figure S1.** The calcium‐activated chloride channel has a very small influence on the action potential shape (blue lines, scale on left axis). The current (black, scale on right axis), however, causes an outward flux during an AP, which leads to long‐term drift (Fig. 2).

**Figure S2.** No alternans were observed in a single cell for a comparable pacing protocol. After pacing, a single cell at 0:5 Hz until steady state was reached, APDs of successive action potentials were measured for each of the indicated BCLs. The 0:5 Hz pacing steady state corresponds to the initial conditions for which alternans were observed in a cable (cf. Fig. 5).

**Figure S3.** Effects of long‐term pacing (see Fig. 2): Model drift upon pacing is also visible at the pacing frequency of 0:5 Hz used by Wang and Sobie (2008), clearly demonstrating that their initial conditions do not represent a steady state.

**Figure S4.** Effects of long‐term pacing (see Fig. 2): The model drift upon pacing is exacerbated at a higher pacing frequency of 10 Hz.

**Figure S5.** Comparison of ionic currents (in pA/pF) for an AP (Fig. 3) elicited after quiescence in a single cell and in tissue. For the fast Na^+^ current, an inset provides a magnified view into the fast dynamics during the upstroke of the AP.

**Figure S6.** Comparison of ionic currents (in pA/pF) during steady‐state APs (Fig. 4) at different BCLs. For the fast Na^+^ current, an inset provides a magnified view into the fast dynamics during the upstroke of the AP.

**Appendix S1.** Model equations.
