# Supplementary figures and images for: Complex restitution behavior and reentry in a cardiac tissue model for neonatal mice
Source: Physiol Rep. 2017 Oct 9;5(19):e13449. doi: 10.14814/phy2.13449 (PMC5641936; doi:10.14814/phy2.13449)

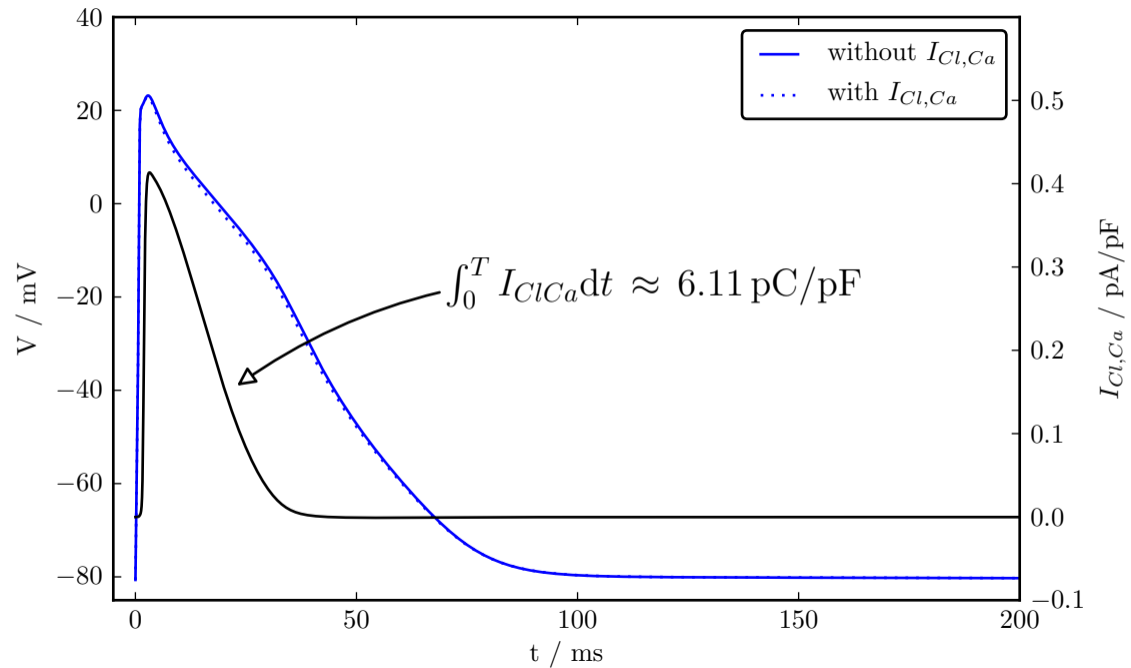

Supplement: Supplementary file 1 — Figure S1. The calcium‐activated chloride channel has a very small influence on the action potential shape (blue lines, scale on left axis). The current (black, scale on right axis), however, causes an outward flux during an AP, which leads to long‐term drift (Fig. 2). [file PHY2-5-e13449-s001.pdf]

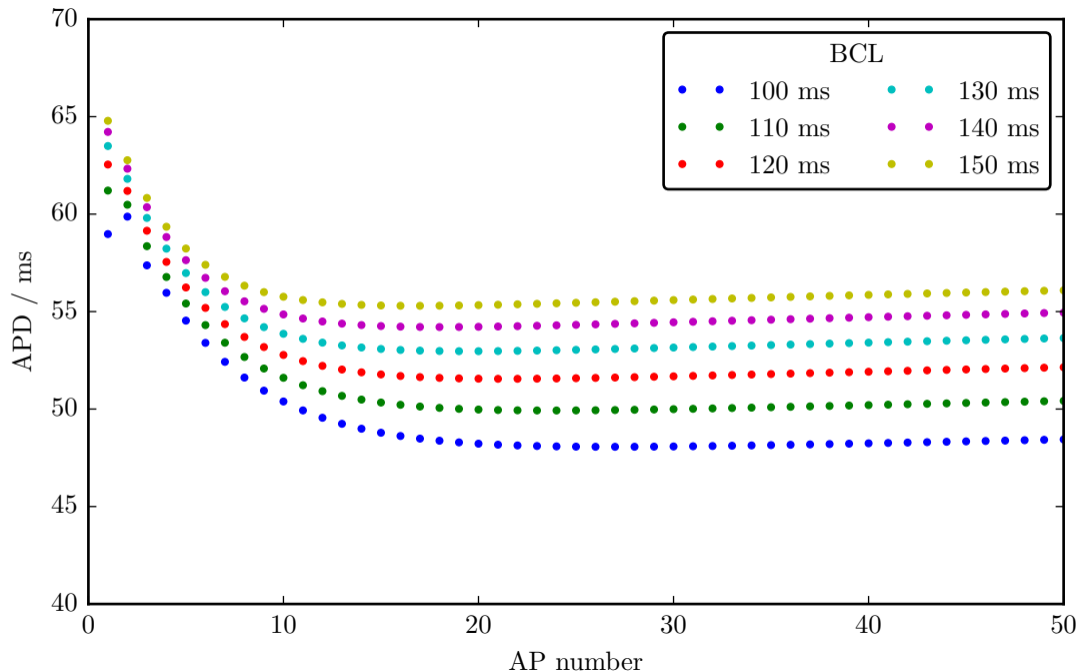

Supplement: Supplementary file 2 — Figure S2. No alternans were observed in a single cell for a comparable pacing protocol. After pacing, a single cell at 0:5 Hz until steady state was reached, APDs of successive action potentials were measured for each of the indicated BCLs. The 0:5 Hz pacing steady state corresponds to the initial conditions for which alternans were observed in a cable (cf. Fig. 5). [file PHY2-5-e13449-s002.pdf]

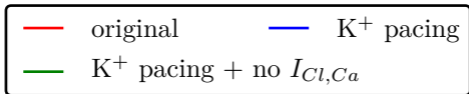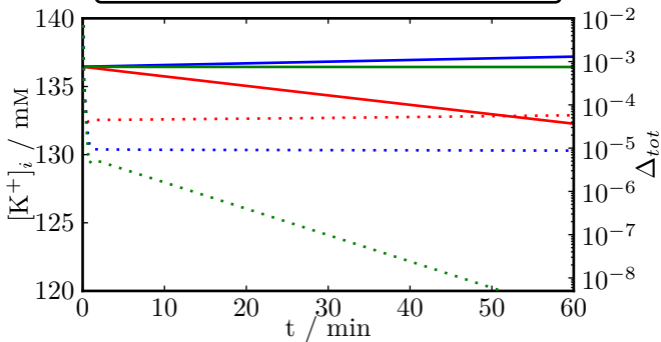

Supplement: Supplementary file 3 — Figure S3. Effects of long‐term pacing (see Fig. 2): Model drift upon pacing is also visible at the pacing frequency of 0:5 Hz used by Wang and Sobie (2008), clearly demonstrating that their initial conditions do not represent a steady state. [file PHY2-5-e13449-s003.pdf]

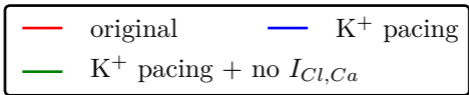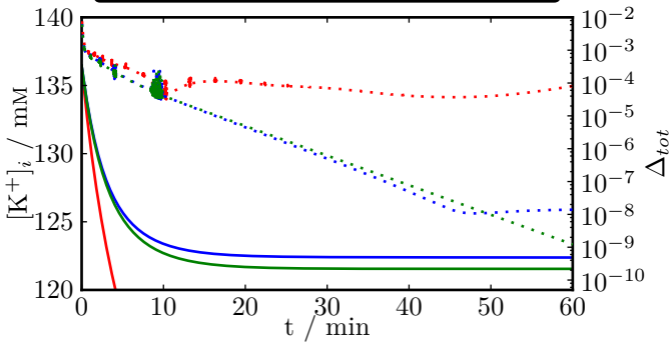

Supplement: Supplementary file 4 — Figure S4. Effects of long‐term pacing (see Fig. 2): The model drift upon pacing is exacerbated at a higher pacing frequency of 10 Hz. [file PHY2-5-e13449-s004.pdf]

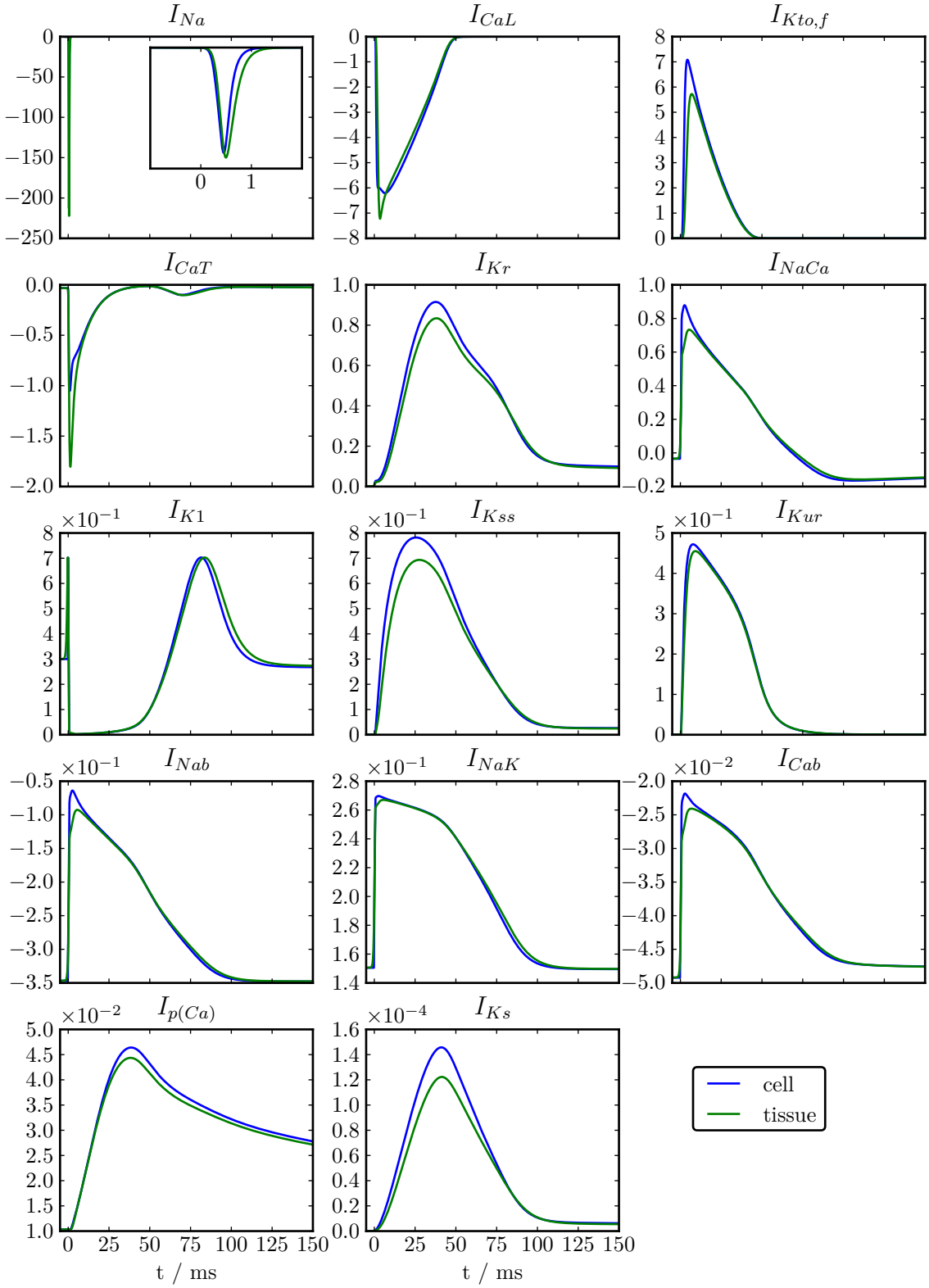

Supplement: Supplementary file 5 — Figure S5. Comparison of ionic currents (in pA/pF) for an AP (Fig. 3) elicited after quiescence in a single cell and in tissue. For the fast Na+ current, an inset provides a magnified view into the fast dynamics during the upstroke of the AP. [file PHY2-5-e13449-s005.pdf]

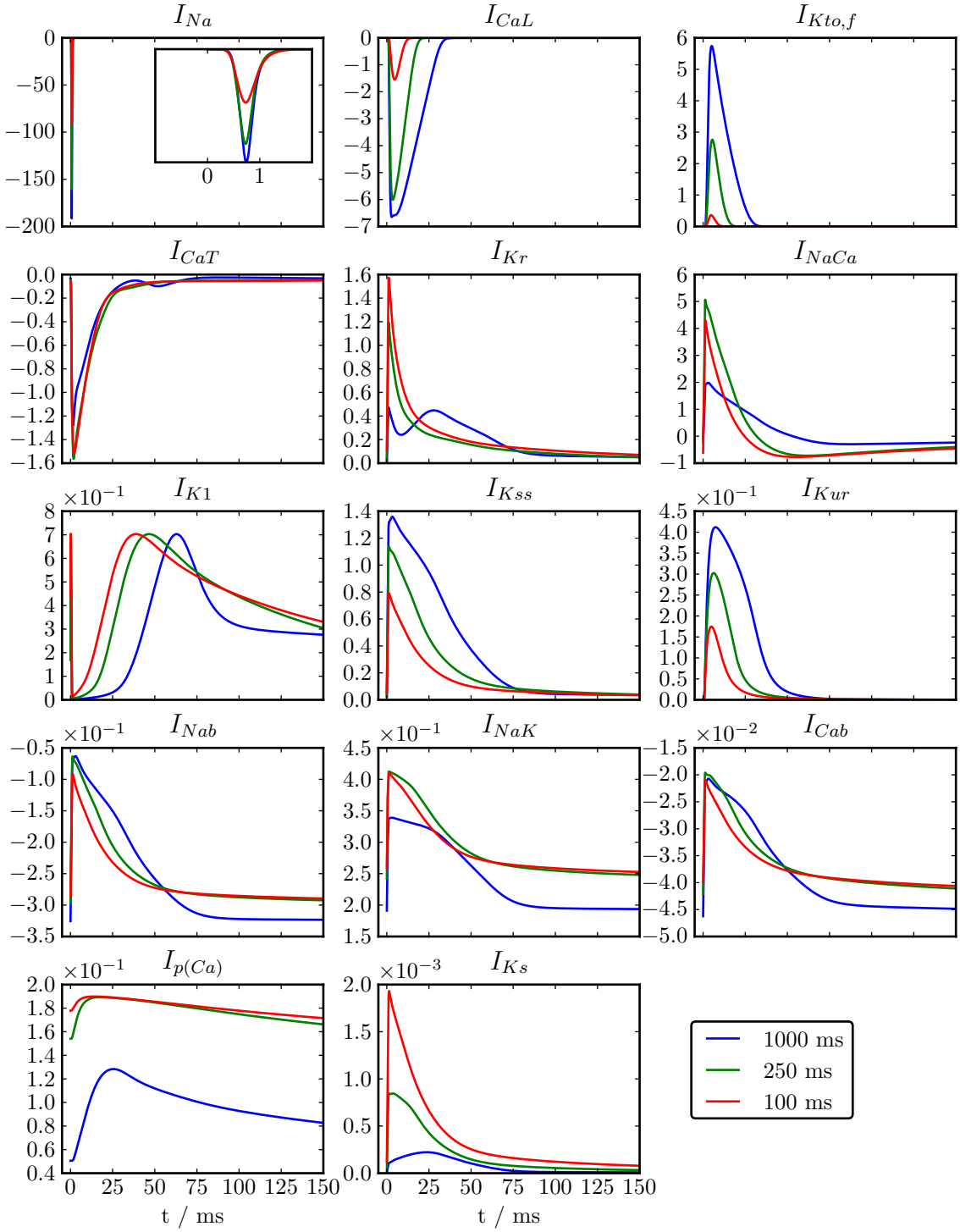

Supplement: Supplementary file 6 — Figure S6. Comparison of ionic currents (in pA/pF) during steady‐state APs (Fig. 4) at different BCLs. For the fast Na+ current, an inset provides a magnified view into the fast dynamics during the upstroke of the AP. [file PHY2-5-e13449-s006.pdf]
