# Supplementary material for: Complex restitution behavior and reentry in a cardiac tissue model for neonatal mice
Source: Physiol Rep. 2017 Oct 9;5(19):e13449. doi: 10.14814/phy2.13449 (PMC5641936; doi:10.14814/phy2.13449)
Supplement: Supplementary file 7 — Appendix S1. Model equations. [file PHY2-5-e13449-s007.pdf]

Appendix S1: Model equations

The full set of equations defining the model of ventricular tissue of neonatal mice (35 ordinary differential equation defining the single cell dynamics and one partial differential equation defining the coupling of cells in tissue) can be found on the following pages. Parameters and initial conditions are given in Table I and Table II. Changes and additional parameters in comparison to the Wang-Sobie model have been highlighted. It should be noted, that there are some small differences between the model definition and parameters in the original publication, the Matlab code provided online by the authors and the CellML description. We have relied on the Matlab code, where the equations differed.

TABLE I: Parameters of the Wang-Sobie model.

name	description	value
R	Ideal gas constant / $\mathrm{Jmol^{-1}K^{-1}}$	8.314
F	Faraday constant / $\mathrm{C\,mmol^{-1}}$	96.5
T	Absolute temperature / K	298
V_{JSR}	Junctional SR volume / µl	1.2e-09
V_{NSR}	Network SR volume / µl	2.098e-07
A_{cap}	Capacitive membrane area / $\mathrm{cm^2}$	1.541e-05
V_{myo}	Myoplasmic volume / µl	2.2826e-06
V_{ss}	Subspace volume / µl	3.0734e-08
C_m	Specific membrane capacitance / $\mathrm{\mu F/cm^2}$	1
$[K^+]_o$	Extracellular K^+ concentration / µM	5400
$[Na^+]_o$	Extracellular Na^+ concentration / µM	140000
$[Ca^{2+}]_o$	Extracellular Ca^{2+} concentration / µM	1000
$[Cl^+]_o$	Extracellular Cl^- concentration / µM	132000
$[Cl^+]_i$	Intracellular Cl^- concentration / µM	30000
G_{Na}	Maximal I_{Na} conductance / mS/µF	10
G_{Nab}	Maximal I_{Nab} conductance / mS/µF	0.0026
G_{Cab}	Maximal I_{Cab} conductance / mS/µF	0.00025
G_{K1}	Maximal I_{K1} conductance / mS/µF	0.235
G_{Kr}	Maximal I_{Kr} conductance / mS/µF	1.17
G_{Ks}	Maximal I_{Ks} conductance / mS/µF	0.046
$G_{Kto,f}$	Maximal $I_{Kto,f}$ conductance / mS/µF	0.1017
G_{Kur}	Maximal I_{Kur} conductance / mS/µF	0.0048

Continued on next page

TABLE I – continued from previous page

name	description	value
G_{Kss}	Maximal I_{Kss} conductance / mS/µF	0.015
k_f	Rate constant for rapid delayed-rectifier K^+ current / ms^{-1}	0.023761
k_b	Rate constant for rapid delayed-rectifier K^+ current / ms^{-1}	0.036778
$P_{Cl,Ca}$	Ca^{2+} activated Cl^- current permeability / cm/s	2.74e-07 / 0.0
$K_{m,Cl}$	Half-activation concentration of Ca^{2+} activated Cl^- current / µM	4
$n_{Cl,Ca}$	Hill exponent of Ca^{2+} activated Cl^- current	3
G_{CaL}	Maximal I_{CaL} conductance / mS/µF	0.19019
E_{CaL}	Reversal potential for L-type Ca^{2+} channel / mV	63
$K_{pc,max}$	Maximal time constant for Ca^{2+} induced inactivation / ms^{-1}	0.23324
$K_{pc,half}$	Half-saturation constant for Ca^{2+} induced inactivation / µM	20
K_{pcb}	Voltage-insensitive rate constant for inactivation / ms^{-1}	0.0005
$I_{CaL,max}$	Normalization constant for L-type Ca^{2+} current / pA/pF	7
G_{CaT}	Maximal I_{CaT} conductance / mS/µF	0.055
E_{CaT}	Reversal potential for T-type Ca^{2+} channel / mV	50
k_{NaCa}	Scaling factor for Na^+/Ca^{2+} exchange / pA/pF	907.68
$K_{m,Na}$	Na^+ half-saturation constant for Na^+/Ca^{2+} exchange / µM	87500
$K_{m,Ca}$	Ca^{2+} half-saturation constant for Na^+/Ca^{2+} exchange / µM	1380
k_{sat}	Na^+/Ca^{2+} exchange saturation factor at very negative potentials	0.1
η	Controls voltage dependence of Na^+/Ca^{2+} exchange	0.35
I_{NaK}^{max}	Maximal Na^+/K^+ exchange current / pA/pF	0.88
$K_{m,Nai}$	Na^+ half-saturation constant for Na^+/K^+ exchange current / µM	21000
$K_{m,Ko}$	K^+ half-saturation constant for Na^+/K^+ exchange current / µM	1500
$I_{p(Ca)}^{max}$	Maximal Ca^{2+} pump current / pA/pF	0.2
$K_{m,p(Ca)}$	Ca^{2+} half-saturation constant for Ca^{2+} pump current / µM	0.5
ν_1	Maximal RyR channel Ca^{2+} permeability / ms^{-1}	0.45
ν_2	Ca^{2+} leak rate constant from the NSR / ms^{-1}	2.088e-05
ν_3	SR Ca^{2+} ATPase maximum pump rate / ms^{-1}	0.09
$K_{m,up}$	Half-saturation constant for SR Ca^{2+} ATPase pump / µM	0.5
τ_{tr}	Time constant for transfer from NSR to JSR / ms	20
τ_{xfer}	Time constant for transfer from subspace to myoplasm / ms	8
k_a^+	RyR P_{C1} - P_{O1} rate constant / $\mu M^{-4} ms^{-1}$	0.00608
k_a^-	RyR P_{O1} - P_{C1} rate constant / ms^{-1}	0.07125
k_b^+	RyR P_{O1} - P_{O2} rate constant / $\mu M^{-3} ms^{-1}$	0.00405

Continued on next page

TABLE I – continued from previous page

name	description	value
k_b^-	RyR P_{O2} - P_{O1} rate constant / ms^{-1}	0.965
k_c^+	RyR P_{O1} - P_{C2} rate constant / ms^{-1}	0.009
k_c^-	RyR P_{C2} - P_{O1} rate constant / ms^{-1}	0.0008
n_{RyR}	RyR Ca^{2+} cooperativity parameter P_{C1} - P_{O1}	4
m_{RyR}	RyR Ca^{2+} cooperativity parameter P_{O1} - P_{O2}	3
$[LTRPN_{tot}]$	Total myoplasmic troponin low-affinity site concentration / µM	35
$[HTRPN_{tot}]$	Total myoplasmic troponin high-affinity site concentration / µM	70
k_{htrpn}^+	Ca^{2+} on rate constant for troponin high-affinity sites / $\mu M^{-1} ms^{-1}$	0.00237
k_{htrpn}^-	Ca^{2+} off rate constant for troponin high-affinity sites / ms^{-1}	3.2e-05
k_{ltrpn}^+	Ca^{2+} on rate constant for troponin low-affinity sites / $\mu M^{-1} ms^{-1}$	0.0327
k_{ltrpn}^-	Ca^{2+} off rate constant for troponin low-affinity sites / ms^{-1}	0.0196
$[CMDN_{tot}]$	Total myoplasmic calmodulin concentration / µM	25
$[CSQN_{tot}]$	Total junctional SR calsequestrin concentration / µM	15000
$K_{m,CMDN}$	Ca^{2+} half-saturation constant for calmodulin / µM	0.238
$K_{m,CSQN}$	Ca^{2+} half-saturation constant for calsequestrin / µM	800
I_{stim}^{max}	maximal stimulus current / pA/pF	-80.0
τ_{stim}	duration of stimulus / ms	0.5
D	Diffusivity (isotropic) / cm^2/ms	0.001

TABLE II: Initial conditions of the model.

name	description	value
V	membrane potential V / mV	-80.6475
$[Ca^{2+}]_i$	Myoplasmic Ca^{2+} concentration / µM	0.2049
$[Ca^{2+}]_{ss}$	Subspace Ca^{2+} concentration / µM	0.1867
$[Ca^{2+}]_{JSR}$	Junctional SR Ca^{2+} concentration / µM	705.542
$[Ca^{2+}]_{NSR}$	Network SR Ca^{2+} concentration / µM	703.306
$[LTRPN_{Ca}]$	Low-affinity troponin-binding sites concentration / µM	8.922
$[HTRPN_{Ca}]$	High-affinity troponin-binding sites concentration / µM	66.0407
$[Na^+]_i$	Intracellular Na^+ concenctration	21747
$[K^+]_i$	Intracellular K^+ concentration	136450
O_L	I_{CaL} channel conducting state	4.4776e-12
C_2	I_{CaL} channel closed state	0.00016996

Continued on next page

TABLE II – continued from previous page		
name	description	value
C_3	I_{CaL} channel closed state	1.1754e-08
C_4	I_{CaL} channel closed state	6.1239e-10
I_1	I_{CaL} channel inactivated state	1.545e-09
I_2	I_{CaL} channel inactivated state	6.4226e-08
I_3	I_{CaL} channel inactivated state	6.5201e-07
P_{C2}	RyR channel closed state	0.1003
P_{O1}	RyR channel opened state	0.0011
P_{O2}	RyR channel opened state	2.9799e-08
P_{RyR}	RyR modulation factor	2.8603e-14
m	I_{Na} channel activation gating variable	0.0032
h	I_{Na} channel inactivation gating variable	0.9606
j	I_{Na} channel slow inactivation gating variable	0.9744
b_T	I_{CaT} current activation gating variable / µM	0.0047
g_T	I_{CaT} current inactivation gating variable / µM	0.902
$a_{to,f}$	$I_{to,f}$ current activation gating variable	0.0032
$i_{to,f}$	$I_{to,f}$ current inactivation gating variable	0.753
n_{Ks}	I_{Ks} current gating variable	0.0026
a_{ur}	I_{Kur} current activation gating variable	0.00052471
i_{ur}	I_{Kur} current inactivation gating variable	0.9866
a_{Kss}	I_{Kss} current gating variable	0.85
C_{K1}	mERG channel closed state	0.0011
C_{K2}	mERG channel closed state	0.00083641
O_K	mERG channel open state	0.0021
I_K	mERG channel inactivated state	0.00043522

Membrane potential

$$-\frac{\mathrm{d}V}{\mathrm{d}t} = I_{ion} + I_{stim} + I_{ec}$$

$$I_{ion} = I_{CaL} + I_{CaT} + I_{p(Ca)} + I_{NaCa} + I_{Cab} + I_{Na} + I_{Nab} + I_{NaK}$$
$$+ I_{Kto,f} + I_{K1} + I_{Ks} + I_{Kur} + I_{Kss} + I_{Kr} + I_{Cl,Ca}$$

Stimulus current

$$I_{stim} = \begin{cases} I_{stim}^{max} & \text{during pulse duration of } \tau_{stim}, \\ 0 & \text{otherwise.} \end{cases}$$

Electrotonic current

$$I_{ec} = -\nabla \cdot \boldsymbol{D} \nabla V$$

$$\boldsymbol{D}_{i,j} = \begin{cases} D, & \text{for } i = j, \\ 0 & \text{otherwise.} \end{cases}$$

Calcium dynamics

Concentration changes in the subcompartments

$$\begin{aligned}
\frac{\mathrm{d}[\mathrm{Ca}^{2+}]_i}{\mathrm{d}t} &= B_i \left(J_{leak} + J_{xfer} - J_{up} - J_{trpn}\right) \\
\frac{\mathrm{d}[\mathrm{Ca}^{2+}]_{ss}}{\mathrm{d}t} &= B_{ss} \Bigg(J_{rel} \frac{V_{JSR}}{V_{ss}} - J_{xfer} \frac{V_{myo}}{V_{ss}} \\
&\quad - \left(I_{CaL} + I_{Cab} + I_{p(Ca)} + I_{CaT} - 2 I_{NaCa}\right) \frac{A_{cap} C_m}{2 V_{ss} F} \Bigg) \\
\frac{\mathrm{d}[\mathrm{Ca}^{2+}]_{JSR}}{\mathrm{d}t} &= B_{JSR} \left(J_{tr} - J_{rel}\right) \\
\frac{\mathrm{d}[\mathrm{Ca}^{2+}]_{NSR}}{\mathrm{d}t} &= \left(J_{up} - J_{leak}\right) \frac{V_{myo}}{V_{NSR}} - J_{tr} \frac{V_{JSR}}{V_{NSR}}
\end{aligned}$$

Buffering

$$\begin{aligned}
B_i &= \left(1 + \frac{[\mathrm{CMDN_{tot}}] K_{m,CMDN}}{\left(K_{m,CMDN} + [\mathrm{Ca}^{2+}]_i\right)^2}\right)^{-1} \\
B_{ss} &= \left(1 + \frac{[\mathrm{CMDN_{tot}}] K_{m,CMDN}}{\left(K_{m,CMDN} + [\mathrm{Ca}^{2+}]_{ss}\right)^2}\right)^{-1} \\
B_{JSR} &= \left(1 + \frac{[\mathrm{CSQN_{tot}}] K_{m,CSQN}}{\left(K_{m,CSQN} + [\mathrm{Ca}^{2+}]_{JSR}\right)^2}\right)^{-1} \\
\frac{\mathrm{d}[\mathrm{LTRPN_{Ca}}]}{\mathrm{d}t} &= k^+_{ltrpn} [\mathrm{Ca}^{2+}]_i \left([\mathrm{LTRPN_{tot}}] - [\mathrm{LTRPN_{Ca}}]\right) - k^-_{ltrpn} [\mathrm{LTRPN_{Ca}}] \\
\frac{\mathrm{d}[\mathrm{HTRPN_{Ca}}]}{\mathrm{d}t} &= k^+_{htrpn} [\mathrm{Ca}^{2+}]_i \left([\mathrm{HTRPN_{tot}}] - [\mathrm{HTRPN_{Ca}}]\right) - k^-_{htrpn} [\mathrm{HTRPN_{Ca}}] \\
J_{trpn} &= \frac{\mathrm{d}[\mathrm{LTRPN_{Ca}}]}{\mathrm{d}t} + \frac{\mathrm{d}[\mathrm{HTRPN_{Ca}}]}{\mathrm{d}t}
\end{aligned}$$

Ca^{2+} fluxes between subcompartments

$$\begin{aligned}
J_{rel} &= \nu_1 \left(P_{O1} + P_{O2}\right) \left([\mathrm{Ca}^{2+}]_{JSR} - [\mathrm{Ca}^{2+}]_{ss}\right) P_{RyR} \\
J_{tr} &= \frac{[\mathrm{Ca}^{2+}]_{NSR} - [\mathrm{Ca}^{2+}]_{JSR}}{\tau_{tr}} \\
J_{xfer} &= \frac{[\mathrm{Ca}^{2+}]_{ss} - [\mathrm{Ca}^{2+}]_i}{\tau_{xfer}} \\
J_{leak} &= \nu_2 \left([\mathrm{Ca}^{2+}]_{NSR} - [\mathrm{Ca}^{2+}]_i\right) \\
J_{up} &= \nu_3 \frac{[\mathrm{Ca}^{2+}]_i^2}{K_{m,up}^2 + [\mathrm{Ca}^{2+}]_i^2}
\end{aligned}$$

Ryanodine receptor (Markov description)

$$\frac{\mathrm{d}P_{RyR}}{\mathrm{d}t} = -0.04 P_{RyR} - \frac{0.1 I_{CaL}}{I_{CaL,max}} e^{\frac{-(V-5)^2}{648}}$$

$$\frac{\mathrm{d}P_{O1}}{\mathrm{d}t} = \left(k_a^+[\mathrm{Ca}^{2+}]_{ss}^{n_{RyR}} P_{C_1} + k_b^- P_{O2} + k_c^- P_{C_2}\right) - \left(k_a^- P_{O1} + k_b^+[\mathrm{Ca}^{2+}]_{ss}^{m_{RyR}} P_{O1} + k_c^+ P_{O1}\right)$$
$$\frac{\mathrm{d}P_{O2}}{\mathrm{d}t} = k_b^+[\mathrm{Ca}^{2+}]_{ss}^{m_{RyR}} P_{O1} - k_b^- P_{O2}$$
$$P_{C_1} = 1 - (P_{C_2} + P_{O1} + P_{O2})$$
$$\frac{\mathrm{d}P_{C_2}}{\mathrm{d}t} = k_c^+ P_{O1} - k_c^- P_{C_2}$$

L-type Ca^{2+} current (Markov description)

$$I_{CaL} = G_{CaL} O \left(V - E_{CaL}\right)$$
$$\frac{\mathrm{d}O}{\mathrm{d}t} = \alpha C_4 + K_{pcb} I_1 + 0.001\left(\alpha I_2 - K_{pcf} O\right) - 4\beta O - \gamma O$$
$$C_1 = 1 - (O + C_2 + C_2 + C_3 + C_4 + I_1 + I_2 + I_3)$$
$$\frac{\mathrm{d}C_3}{\mathrm{d}t} = 3\alpha C_2 + 3\beta C_4 - 2\beta C_3 - 2\alpha C_3$$
$$\frac{\mathrm{d}C_4}{\mathrm{d}t} = 2\alpha C_3 + 4\beta O + 0.01\left(4K_{pcb}\beta I_1 - \alpha\gamma C_4\right) + 0.002\left(4\beta I_2 - K_{pcf} C_4\right) + 4\beta K_{pcb} I_3 - 3\beta C_4 - \alpha C_4 - 1\gamma K_{pcf} C_4$$
$$\frac{\mathrm{d}I_1}{\mathrm{d}t} = \gamma O + 0.001\left(\alpha I_3 - K_{pcf} I_1\right) + 0.01\left(\alpha\gamma C_4 - 4\beta K_{pcf} I_1\right) - K_{pcb} I_1$$
$$\frac{\mathrm{d}I_2}{\mathrm{d}t} = 0.001\left(K_{pcf} O - \alpha I_2\right) + K_{pcb} I_3 + 0.002\left(K_{pcf} C_4 - 4\beta I_2\right) - \gamma I_2$$
$$\frac{\mathrm{d}I_3}{\mathrm{d}t} = 0.001\left(K_{pcf} I_1 - \alpha I_3\right) + \gamma I_2 + 1\gamma K_{pcf} C_4 - 4\beta K_{pcb} I_3 - K_{pcb} I_3$$
$$\alpha = 0.4e^{\frac{(V+12)}{10}} \frac{1 + 0.7e^{\frac{-(V+40)^2}{10}} - 0.75e^{\frac{-(V+20)^2}{400}}}{1 + 0.12e^{\frac{(V+12)}{10}}}$$
$$\beta = 0.05e^{\frac{-(V+12)}{13}}$$
$$\gamma = \frac{K_{pc,max}[\mathrm{Ca}^{2+}]_{ss}}{K_{pc,half} + [\mathrm{Ca}^{2+}]_{ss}}$$
$$K_{pcf} = 13\left(1 - e^{\frac{-(V+14.5)^2)}{100}}\right)$$

T-type Ca^{2+} current (Hodgkin-Huxley description)

$$I_{CaT} = G_{CaT} b g \left(V - E_{CaT}\right)$$
$$\frac{\mathrm{d}b}{\mathrm{d}t} = \frac{b_\infty - b}{\tau_b} \qquad \frac{\mathrm{d}g}{\mathrm{d}t} = \frac{g_\infty - g}{\tau_g}$$
$$b_\infty = \frac{1}{1 + e^{\frac{-(V+48)}{6.1}}} \qquad g_\infty = \frac{1}{1 + e^{\frac{(V+66)}{6.6}}}$$
$$\tau_b = 0.1 + \frac{5.4}{1 + e^{\frac{(V+100)}{6.6}}} \qquad \tau_g = 8 + \frac{32}{1 + e^{\frac{(V+65)}{5}}}$$

Ca^{2+} pump current

$$I_{p(Ca)} = I_{p(Ca)}^{max} \frac{[\mathrm{Ca}^{2+}]_i^2}{K_{m,p(Ca)}^2 + [\mathrm{Ca}^{2+}]_i^2}$$

Na^{+}-Ca^{2+} exchange current

$$I_{NaCa} = k_{NaCa} \frac{1}{K_{m,Na}^3 + [\mathrm{Na}^+]_o^3} \frac{1}{K_{m,Ca} + [\mathrm{Ca}^{2+}]_o} \frac{1}{\left(1 + k_{sat} e^{\frac{(\eta-1)VF}{RT}}\right)} \cdot \left(e^{\frac{\eta VF}{RT}} [\mathrm{Na}^+]_i^3 [\mathrm{Ca}^{2+}]_o - e^{\frac{(\eta-1)VF}{RT}} [\mathrm{Na}^+]_o^3 [\mathrm{Ca}^{2+}]_i\right)$$

Ca^{2+} background current

$$I_{Cab} = G_{Cab}\left(V - E_{CaN}\right)$$

$$E_{CaN} = \frac{RT}{2F}\ln\frac{[\mathrm{Ca}^{2+}]_o}{[\mathrm{Ca}^{2+}]_i}$$

Sodium dynamics

Concentration change

$$\frac{\mathrm{d}[\mathrm{Na}^+]_i}{\mathrm{d}t} = -\left(I_{Na} + I_{Nab} + 3I_{NaK} + 3I_{NaCa}\right)\frac{A_{cap}C_m}{V_{myo}F}$$

$$E_{Na} = \frac{RT}{F}\ln\frac{[\mathrm{Na}^+]_o}{[\mathrm{Na}^+]_i}$$

Fast Na^+ current (Hodgkin-Huxley description)

$$I_{Na} = G_{Na}m^3hj\left(V - E_{Na}\right)$$

$$\frac{\mathrm{d}m}{\mathrm{d}t} = \alpha_m - \left(\alpha_m + \beta_m\right)m \qquad \frac{\mathrm{d}h}{\mathrm{d}t} = \alpha_h - \left(\alpha_h + \beta_h\right)h$$

$$\alpha_m = \frac{0.32\left(V + 47.13\right)}{1 - e^{-0.1(V+47.13)}} \qquad \alpha_h = \begin{cases} 0.135e^{\frac{(80+V)}{-6.8}} & \text{if } V < -40, \\ 0 & \text{otherwise.} \end{cases}$$

$$\beta_m = 0.08e^{\frac{-V}{11}} \qquad \beta_h = \begin{cases} 3.56e^{0.079V} + 310000e^{0.35V} & \text{if } V < -40, \\ \frac{1}{0.13\left(1+e^{\frac{V+10.66)}{-11.1}}\right)} & \text{otherwise.} \end{cases}$$

$$\frac{\mathrm{d}j}{\mathrm{d}t} = \alpha_j - \left(\alpha_j + \beta_j\right)j$$

$$\alpha_j = \begin{cases} \frac{-\left(127140e^{0.2444V}+3.474\cdot 10^{-5}e^{-0.04391V}\right)(V+37.78)}{1+e^{0.311(V+79.23)}} & \text{if } V < -40, \\ 0 & \text{otherwise.} \end{cases}$$

$$\beta_j = \begin{cases} \frac{0.1212e^{-0.01052V}}{1+e^{-0.1378(V+40.14)}} & \text{if } V < -40, \\ \frac{0.3e^{-2.535\cdot 10^{-7}V}}{1+e^{-0.1(V+32)}} & \text{otherwise.} \end{cases}$$

Na^+ background current

$$I_{Nab} = G_{Nab}\left(V - E_{Na}\right)$$

Potassium dynamics

Concentration change

$$\frac{\mathrm{d}[\mathrm{K}^+]_i}{\mathrm{d}t} = \left(-I_{Kto,f} - I_{K1} - I_{Ks} - I_{Kss} - I_{Kur} - I_{Kr} + 2I_{NaK} - I_{stim} - I_{ec}\right)\frac{A_{cap}C_m}{V_{myo}F}$$

$$E_K = \frac{RT}{F}\ln\frac{[\mathrm{K}^+]_o}{[\mathrm{K}^+]_i}$$

Fast transient outward K$^+$ current (Hodgkin-Huxley description)

$$I_{Kto,f} = G_{Kto,f} a_{to,f}^{6.5} i_{to,f} (V - E_K)$$

$$\frac{\mathrm{d}a_{to,f}}{\mathrm{d}t} = \alpha_a (1 - a_{to,f}) - \beta_a a_{to,f} \qquad \frac{\mathrm{d}i_{to,f}}{\mathrm{d}t} = \frac{i_{tof,\infty} - i_{to,f}}{\tau_{itof}}$$

$$\alpha_a = 0.18064 e^{0.03577(V+30)} \qquad \alpha_i = \frac{0.000152 e^{\frac{-(V-3.81)}{15.75}}}{1 + 0.0067083 e^{\frac{-(V+132.05)}{15.75}}}$$

$$\beta_a = 0.3956 e^{-0.06237(V+30)} \qquad \beta_i = \frac{0.00095 e^{\frac{V+132.05}{15.75}}}{1 + 0.051335 e^{\frac{V+132.05}{15.75}}}$$

$$\tau_{itof} = \left(\frac{0.000152 e^{\frac{-(V+13.5)}{7}}}{1 + 0.067083 e^{\frac{-(V+33.5)}{7}}} + \frac{0.00095 e^{\frac{V+33.5}{7}}}{1 + 0.051335 e^{\frac{V+33.5}{7}}} \right)^{-1} \qquad i_{tof,\infty} = \frac{\alpha_i}{\alpha_i + \beta_i}$$

Ultrarapidly activating delayed-rectifier K$^+$ current (Hodgkin-Huxley description)

$$I_{Kur} = G_{Kur} a_{ur} i_{ur} (V - E_K)$$

$$\frac{\mathrm{d}a_{ur}}{\mathrm{d}t} = \frac{a_{ur,\infty} - a_{ur}}{\tau_{aur}} \qquad \frac{\mathrm{d}i_{ur}}{\mathrm{d}t} = \frac{i_{ur,\infty} - i_{ur}}{\tau_{iur}}$$

$$a_{ur,\infty} = \left(1 + e^{\frac{-(V+22.5)}{7.7}}\right)^{-1} \qquad i_{ur,\infty} = \left(1 + e^{\frac{V+45.2}{5.7}}\right)^{-1}$$

$$\tau_{aur} = 0.493 e^{-0.0629V} + 2.058 \qquad \tau_{iur} = 1200 - \frac{170}{1 + e^{\frac{V+45.2}{5.7}}}$$

Noninactivating steady-state K$^+$ current (Hodgkin-Huxley description)

$$I_{Kss} = G_{Kss} a_{Kss} (V - E_K)$$

$$\frac{\mathrm{d}a_{Kss}}{\mathrm{d}t} = \frac{a_{ur,\infty} - a_{Kss}}{\tau_{Kss}} \qquad \tau_{Kss} = 39.3 e^{-0.0862V} + 13.17$$

Time-independent K$^+$ current

$$I_{K1} = G_{K1} \frac{[\mathrm{K}^+]_o}{[\mathrm{K}^+]_o + 210} \frac{V - E_K}{1 + e^{0.0896(V - E_K)}}$$

Slow delayed-rectifier K$^+$ current (Hodgkin-Huxley description)

$$I_{Ks} = G_{Ks} n_{Ks}^2 (V - E_K)$$

$$\frac{\mathrm{d}n_{Ks}}{\mathrm{d}t} = \alpha_n (1 - n_{Ks}) - \beta_n n_{Ks}$$

$$\alpha_n = \frac{4.81333 \cdot 10^{-6} (V + 26.5)}{1 - e^{-0.128(V+26.5)}} \qquad \beta_n = 9.53333 \cdot 10^{-5} e^{-0.038(V+26.5)}$$

Rapid delayed-rectifier K$^+$ current (Markov description)

$$I_{Kr} = G_{Kr} O_K (V - E_K)$$

$$\frac{\mathrm{d}O_K}{\mathrm{d}t} = \alpha_{a1} C_{K2} + \beta_i I_K - \beta_{a1} O_K - \alpha_i O_K$$

$$C_{K0} = 1 - (C_{K1} + C_{K2} + O_K + I_K)$$

$$\frac{\mathrm{d}C_{K2}}{\mathrm{d}t} = k_f C_{K1} + \beta_{a1} O_K - k_b C_{K2} - \alpha_{a1} C_{K2}$$

$$\frac{\mathrm{d}C_{K1}}{\mathrm{d}t} = \alpha_{a0} C_{K0} + k_b C_{K2} - \beta_{a0} C_{K1} - k_f C_{K1}$$

$$\frac{\mathrm{d}I_K}{\mathrm{d}t} = \alpha_i O_K - \beta_i I_K$$

$$\alpha_{a0} = 0.022348e^{0.01176V} \qquad \beta_{a0} = 0.047002e^{-0.0631V}$$

$$\alpha_{a1} = 0.013733e^{0.038198V} \qquad \beta_{a1} = 0.0000689e^{-0.04178V}$$

$$\alpha_{i} = 0.090821e^{0.023391(V+5)} \qquad \beta_{i} = 0.006497e^{-0.03268(V+5)}$$

Na^{+}-K^{+} pump current

$$I_{NaK} = I_{NaK}^{max} \frac{f_{NaK}}{1 + \left(\frac{K_{m,Nai}}{[\mathrm{Na}^+]_i}\right)^{1.5}} \frac{[\mathrm{K}^+]_o}{[\mathrm{K}^+]_o + K_{m,Ko}}$$

$$f_{NaK} = \left(1 + 0.1245e^{\frac{-0.1VF}{RT}} + 0.0365\sigma e^{\frac{-VF}{RT}}\right)^{-1}$$

$$\sigma = \frac{1}{7}\left(e^{\frac{[\mathrm{Na}^+]_o}{67300}} - 1\right)$$

Chloride dynamics

Ca^{2+}-activated Cl^{-} current, not active ($P_{Cl,Ca} = 0$) except stated otherwise

$$I_{Cl,Ca} = P_{Cl,Ca} f_{Cl,Ca} \frac{VF^2}{RT} \frac{[\mathrm{Cl}^+]_o e^{\frac{VF}{RT}} - [\mathrm{Cl}^+]_i}{e^{\frac{VF}{RT}} - 1} \qquad f_{Cl,Ca} = \left(\frac{[\mathrm{Ca}^{2+}]_{ss}}{(K_{m,Cl} + [\mathrm{Ca}^{2+}]_{ss})}\right)^{n_{Cl,Ca}}$$
